# Quinolone resistance mutations in the faecal microbiota of Swedish travellers to India

**DOI:** 10.1186/s12866-015-0574-6

**Published:** 2015-10-24

**Authors:** Anna Johnning, Erik Kristiansson, Martin Angelin, Nachiket Marathe, Yogesh S. Shouche, Anders Johansson, D. G. Joakim Larsson

**Affiliations:** Department of Infectious Diseases, Institute of Biomedicine, University of Gothenburg, Guldhedsgatan 10, SE-413 46 Gothenburg, Sweden; Department of Mathematical Sciences, Chalmers University of Technology, SE-412 96 Gothenburg, Sweden; Department of Clinical Microbiology, Infectious Diseases, Umeå University, SE-901 85 Umeå, Sweden; Microbial Culture Collection, National Centre for Cell Science, Ganeshkhind, Pune 411 007 India; Laboratory for Molecular Infection Medicine Sweden, Department of Clinical Microbiology, Bacteriology, Umeå University, SE- 901 87 Umeå, Sweden

**Keywords:** Antimicrobial resistance, Gut microbiota, 454 sequencing, Fluoroquinolones

## Abstract

**Background:**

International travel contributes to the spread of antibiotic resistant bacteria over the world. Most studies addressing travel-related changes in the faecal flora have focused on specific mobile resistance genes, or depended on culturing of individual bacterial isolates. Antibiotic resistance can, however, also spread via travellers colonized by bacteria carrying chromosomal antibiotic resistance mutations, but this has received little attention so far. Here we aimed at exploring the abundance of chromosomal quinolone resistance mutations in *Escherichia* communities residing in the gut of Swedish travellers, and to determine potential changes after visiting India. Sweden is a country with a comparably low degree of quinolone use and quinolone resistance, whereas the opposite is true for India.

**Methods:**

Massively parallel amplicon sequencing targeting the quinolone-resistance determining region of *gyrA* and *parC* was applied to total DNA extracted from faecal samples. Paired samples were collected from 12 Swedish medical students before and after a 4–15 week visit to India. Twelve Indian residents were included for additional comparisons. Methods known resistance mutations were common in Swedes before travel as well as in Indians, with a trend for all mutations to be more common in the Indian sub group. There was a significant increase in the abundance of the most common amino acid substitution in GyrA (S83L, from 44 to 72 %, *p* = 0.036) in the samples collected after return to Sweden. No other substitution, including others commonly associated with quinolone resistance (D87N in GyrA, S80I in ParC) changed significantly. The number of distinct genotypes encoded in each traveller was significantly reduced after their visit to India for both GyrA (*p* = 0.0020) and ParC (*p* = 0.0051), indicating a reduced genetic diversity, similar to that found in the Indians.

**Conclusions:**

International travel can alter the composition of the *Escherichia* communities in the faecal flora, favouring bacteria carrying certain resistance mutations, and, thereby, contributes to the global spread of antibiotic resistance. A high abundance of specific mutations in Swedish travellers before visiting India is consistent with the hypothesis that these mutation have no fitness cost even in the absence of an antibiotic selection pressure.

**Electronic supplementary material:**

The online version of this article (doi:10.1186/s12866-015-0574-6) contains supplementary material, which is available to authorized users.

## Background

Bacterial antibiotic resistance is one of the greatest threats to public health globally. Bacteria can acquire resistance by gaining mobile antibiotic resistance genes or by *de novo* mutations in pre-existing DNA. International travel has been identified as one of the facilitators of the global spread of resistant bacteria, together with, e.g., trade and migration [1, 2]. Indeed, in both population-based case-control studies [3] and time series studies [4, 5] using classical culturing of bacteria, it has been shown that international travel is associated with an increased abundance of resistant bacteria in the gut flora. Studies utilising PCR to assess the presence of resistance genes in gut microbiota have demonstrated the acquisition of such genes after international travel [6–8]. However, little is known about how travel affects the relative abundance of resistance mutations in human microbiota.

Fluoroquinolone antibiotics are a clinically important class of broad-spectrum antibiotics to which bacteria have acquired a high frequency of resistance in many parts of the world [9]. Fluoroquinolones inhibit type II topoisomerases, DNA gyrase and topoisomerase VI, which are enzymes that introduce double-stranded breaks in DNA to maintain DNA topology during replication and transcription. Both enzymes are tetramers (X_2_Y_2_), where DNA gyrase encoded by genes *gyrA* and *gyrB,* and topoisomerase IV is encoded by *parC* and *parE*. Resistance against fluoroquinolones is caused by either chromosomal mutations in the quinolone-resistance determining region (QRDR) of the target genes, especially in *gyrA* and *parC* [10], or by mobile resistance genes (*qnr*, *qepA*, *oqxAB*, or *aac(6′)-Ib-cr*) [11]. Plasmid-mediated quinolone resistance, however, usually confers a lower level of resistance than chromosomal mutations. A study of fluoroquinolone-resistant clinical *E. coli* isolates in Houston, TX, USA, showed that all isolates had one or several amino acid substitutions in GyrA, and 85 % of these isolates had additional substitutions in ParC [12]. The gene *aac(6′)-lb-cr* was only detected in isolates that also possessed chromosomal mutations, and none of the studied isolates carried *qnrA*. These results underline that chromosomal mutations in *gyrA* and *parC* are the main causes of clinical resistance to fluoroquinolones.

In Sweden fluoroquinolones constituted 5.9 % of the total consumption of antibacterials for systemic use in 2010 (0.77 and 0.164 Defined Daily Doses, DDD, per 1 000 inhabitants and day in the primary care sector and the hospital sector, respectively) [13], and in 2011, 7.9 % of all tested invasive *E. coli* infections were fluoroquinolone resistant [14]. During the same years, the average consumption of fluoroquinolones in the primary care sector in Europe was 1.44 DDD per 1 000 inhabitants and year (excluding countries only reporting total sales), and the average frequency of resistance in invasive *E. coli* was 23 %. Sweden is, therefore, a country with both a low fluoroquinolone consumption and a low fluoroquinolone resistance burden. Although India lacks a national surveillance system for both antibiotic consumption and resistance, there are reports from some areas. When antibiotic usage in private clinics in New Delhi, India, was studied in 2008, approximately 33 % of the consumed DDD of antibiotics were fluoroquinolones (estimated 74 DDD per 1 000 patients and day) [15]. Furthermore, a study of resistance in uropathogens isolated in a tertiary-care hospital in the south of India found that 73.1 % of the *E. coli* isolates were ciprofloxacin resistant [16]. Hence, both fluoroquinolone consumption and resistance levels in, at least, bacteria causing urinary tract infections are higher in parts of India compared to Sweden. The difference in antibiotic resistance burden is also supported by findings of the spread of mobile resistance genes from India to Sweden. For example, the metallo-β-lactamase NDM-1 was isolated from a Swedish patient returning from India [17]. Moreover, a prospective study of Swedish travellers found that, out of the studied countries, India was the country associated with the highest risk of acquiring CTX-M-type extended spectrum β-lactamases (ESBLs) to the gut (88 % of travellers visiting India) [18]. A study of Swedish patients suffering from traveller’s diarrhoea showed that 79 % of travellers to India, but only 3 % of patients travelling within Europe, had ESBL-producing bacteria in their stool [8]. Lastly, travel from Sweden to the Indian subcontinent has been associated with a significantly increased risk of acquiring ESBL-producing Enterobacteriaceae into the faecal flora [5].

In this study, we assessed the effect of travelling from Sweden to India on the abundance of quinolone resistance mutations in faecal *Escherichia* communities. Massively parallel amplicon sequencing of metagenomic DNA isolated from paired samples collected from Swedes before and after their visit to India, was used to determine the sequence of the QRDR of *gyrA* and *parC*. By using this method, we could study resistance mutations in an bacterial community without relying on culturing of individual bacteria. Samples from an Indian sub-population were also included and provided additional references.

## Methods

Paired faecal samples were collected from 12 Swedish students from Universities in Umeå, Stockholm, and Gothenburg between December 2010 and February 2013, before and after their visit to India for internship or study at hospitals. The volunteers were 20–30 years old, 75 % were female, and the duration of the visit was 28–106 days, during which 67 % of the participants had regular contact with patients. Samples were collected 1–77 days before departure (on average, 13 days) and 2–44 days after returning to Sweden (on average, 22 days) (see Additional file 1: Table S1 and S2). The samples were collected by the volunteers themselves at home, sent to the lab in sterile tubes, and stored at −20 °C upon arrival the following day. We also had access to faecal samples collected from native Indians living in a rural area near Hyderabad. Despite them not being matched to the Swedish participants, 12 of these samples were included in this study for additional comparisons. The Indian sampling was carried out in July 2010, the age span of the participants was 12–40 years, and 33 % were female (see Additional file 1: Table S1). We have previously studied environmental fluoroquinolone pollution near Hyderabad and, therefore, can state that the Indian participants all lived in villages where no fluoroquinolones have been detected in either well water or soil [19]. None of the volunteers in the study, neither Swedes nor Indians, had taken any antibiotics within six months prior to the sampling. The Swedish sampling was approved by the regional ethical review board in Umeå, Sweden (2011-357-32M), and institutional ethical clearance (IEC, National Centre for Cell Science, Pune, India) was obtained for the Indian sampling. Written informed consent was obtained from all participants. DNA extraction was performed using the QIAamp® DNA Stool Mini Kit (QIAGEN) according to the manufacturer’s protocol. The DNA concentrations were measured using a NanoDrop, and the samples were stored at −20 °C before PCR amplification of the target genes.

Using Primer3Plus, primers were designed targeting the QRDR of *gyrA* and *parC*. The QRDR was defined as the region of each gene including all resistance mutations in *E. coli* reported by Ruiz [20] and was given as the target region in Primer3Plus. To capture as large a proportion of the *Escherichia* community as possible, all *Escherichia* sequences annotated as *gyrA* or *parC* were downloaded from Pathosystems Resource Integration Center (PATRIC) [21], and MUSCLE [22] was used to produce one multiple sequence alignment for each gene. Because the *Shigella* genus is indistinguishable from *Escherichia* in nucleotide sequence in the target region of both *gyrA* and *parC*, all *Shigella gyrA* and *parC* sequences in PATRIC were also added to the multiple sequence alignment. Variable positions in the alignments were given as excluded regions in Primer3Plus, and the product size range was set to 250–350 bp, close to the average length of a 454 sequence read. The top ten primer pairs for each gene suggested by the software were tested experimentally, and the primer pair producing most amplicons with the expected target length, as determined using gel electrophoresis, was selected for the study. The selected primer sequences, target region, expected target size, and annealing temperature are listed in Table 1.

**Table 1:** Selected primers with target regions and experimental setup

Gene	Target region	Product size	Forward primer	Reverse primer	Annealing T
*gyrA*	129–439	311 bp	ggtacaccgtcgcgtacttt	caacgaaatcgaccgtctct	57 °C
*parC*	20–306	287 bp	gccttgcgctacatgaattt	accatcaaccagcggataac	57 °C

The PCR amplification of QRDRs was performed as follows: 1xGotaq®Reaction buffer, 0.2 mM dNTP Mix, 0.4 μM forward primer, 0.4 reverse primer, 1.25u GoTaq® DNA polymerase (Promega Corporation), 50 ng template, and water up to 25 μl were mixed in sterile 0.2 μg tubes. The PCR amplification included an initial denaturation at 95 °C for 2 min, denaturation at 95 °C for 10 s, annealing at 57–61 °C for 30 s, extension at 72 °C for 1 min, and a final extension for 7 min. To ensure a sufficient yield of amplicon DNA, PCR was run for 40 cycles. The PCR products were separated using 1.5 % agarose gel electrophoresis and visualised with GelRed. The DNA fragments matching the target length were excised from the gel, placed in a microcentrifuge tube, and extracted using the GeneJET™ gel extraction kit (Fermentas International Inc.) according to the manufacturer’s protocol. Purified *gyrA* and *parC* amplicons originating from the same faecal sample were pooled and stored at −20 °C before sequencing.

GATC Biotech (Konstanz, Germany) performed the massively parallel pyrosequencing with the GS FLX System using Titanium chemistry. Tagged adaptor sequences were added to the amplicons using titanium chemistry and fusion primers before all samples were pooled and sequenced on one full plate. GS Amplicon Variant Analyzer from 454 (version 2.5p1) was used to align the de-multiplexed data onto reference gene sequence of *gyrA* and *parC* belonging to *E. coli* K-12 MG1655 [RefSeq: NC_000913.3]. The multiple alignments were exported in FASTA format, producing one alignment file for each gene and sample. To remove frameshifts caused by homopolymer sequencing errors, any positions in the multiple alignments having a gap in the reference sequence were removed. Any remaining gaps in the read sequences were substituted with the reference sequence in the corresponding alignment position. The resulting gapless reads were translated using the EMBOSS tool Transeq [23] with the bacterial codon table (*gyrA*: reading frame 1, *parC*: reading frame 2). To remove reads from untargeted genera, all sequences annotated with gene symbol *gyrA* or *parC* were downloaded from the Comprehensive Microbial Resource (CMR) [24] and matched to all reads using BLASTn [25]. Only reads with their best hit to *Escherichia* or *Shigella* and with E-values less than 1 × 10^−100^ were used in this study; this reduced the risk of interpreting interspecies variability as substitutions in the targeted genera. The number of observed genotypes in a sample was measured as the number of unique amino acid sequences detected in more than 0.5 % of the total number of analysed reads in the sample. Differences between the Swedish samples taken before travel to India and the samples taken after return were tested using a paired t-test with a two-tailed distribution. The Swedish samples taken before travel and the Indian samples were compared using a two-sample t-test with a two-tailed distribution assuming equal variance.

## Results

The sequencing produced 291 × 10^6^ bp distributed over almost one million reads (on average, 24 692 reads per sample). The alignment of the reads matched 38 and 58 % of all reads onto the *gyrA* and *parC* reference sequences from *E. coli* K-12 MG1566, respectively (4 % did not align to either). After discarding reads that were too dissimilar from *Escherichia* and *Shigella* reference sequences, 53 % of the reads aligned to *gyrA* remained for further analysis, and 96 % of the reads aligned to *parC* (Additional file 1: Table S1). Due to high frequency of reads matching *Citrobacter*, the loss of data in the filtering step was higher for the *gyrA* data compared to the *parC* data. This implies that the *parC* primers are more specific for the genus *Escherichia*.

**Fig. 1:**
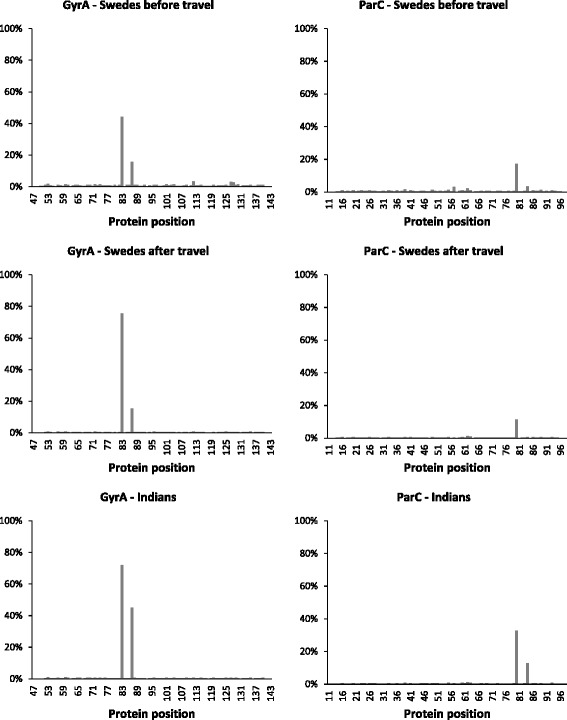
Average abundance of amino acid substitutions in the quinolone resistance determining region. Each chart represents a study group (Swedes before travel, Swedes after travel, and Indians) and a targeted sequence (GyrA and ParC). The protein sequences of GyrA and ParC in E. coli K-12 MG1655 [RefSeq: NC_000913.3] was used as a reference

**Fig. 2:**
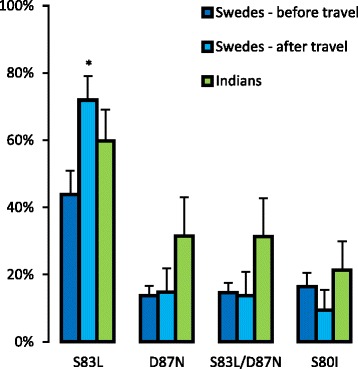
Average abundance of the most commonly detected substitutions. All substitutions detected in >5 % of all analysed reads are included. For the quinolone resistance determining region of GyrA, this was the substitutions S83L, D87N, and the combination thereof. In ParC it was the S80I substitution. Error bars indicates the standard error of the mean, and an asterisk indicates significant difference compared to the Swedish samples collected before travel (*p* < 0.05)

The abundance of amino acid substitutions was recorded in each sample by comparing translated read sequences to the reference peptide sequence. The average substitution abundance in each sampled group, and for each gene, is illustrated in Fig. 1. In GyrA, amino acids S 83 and D 87 where the most commonly substituted amino acids, and S83L and D87N where the most detected substitutions. Of all the reads encoding the D87N substitution, 98 % also encoded the S83L substitution. There was a significant increase (0.28 increase in relative abundance, *p* = 0.036) in the abundance of the most commonly detected substitution, S83L, when comparing the Swedish samples collected before travel to India to the samples taken after return to Sweden (Fig. 2). In the ParC protein, amino acid S 80 was the most commonly substituted, with substitution S80I being the most detected substitution. No other substitutions occurred in more than 5 % of all the analysed reads, but all the substitutions detected in more than 1 % of all reads are listed in the Additional file 1: Table S3. There were no significant differences between the Indian samples and the Swedish samples taken before travel in terms of the abundance of the common substitutions (*p* > 0.05).

**Fig. 3:**
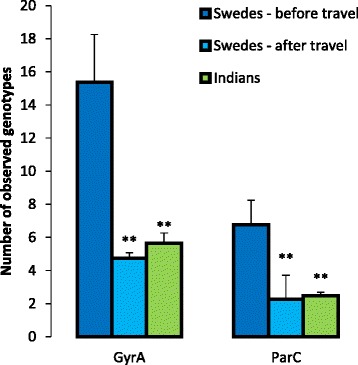
Number of observed genotypes of the quinolone resistance determining regions in GyrA and ParC. Only genotypes detected in > 0.5 % of the analysed reads in each sample are included. Error bars indicates the standard error of the mean, and asterisks indicates a significant difference compared to the Swedish samples collected before travel (*p* < 0.01)

The Swedish samples collected before travel to India showed a higher number of observed genotypes compared to both the samples collected after return (GyrA *p* = 0.00197, ParC *p* = 0.00506), and the Indian samples (GyrA *p* = 0.0016, ParC *p* = 0.0045) (Fig. 3). There was, however, no difference between the Swedish post-travel samples and the Indian samples with regard to the number of genotypes.

**Fig. 4:**
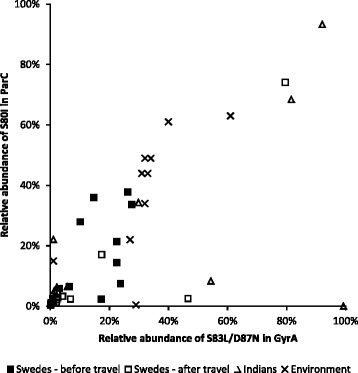
Correlation between the most common substitutions in GyrA and ParC. The relative abundance of the double substitution of S83L and D87N in GyrA, and the S80I substitution in ParC in faecal and environmental (Johnning et al., under review) *Escherichia communities*

To investigate the possible co-occurrence of *gyrA* and *parC* mutations at the community level, their relative abundances were illustrated in a scatter plot (Fig. 4). The correlation between the abundance of the double substitution of S83L and D87N in GyrA and the S80I substitution in ParC was high (sample correlation coefficient 0.686). To determine whether this correlation holds for other *Escherichia* communities, environmental data were added to the scatter plot. The data were generated using the same primers and analyses described in the current study but using sediment samples (Johnning et al. unpublished), and the same pattern was observed.

## Discussion

For some types of antibiotics, including fluoroquinolones, chromosomal mutations play a more important role than horizontally transferrable resistance genes in the development of clinically resistant pathogens [12]. Here, we have determined how the abundance of chromosomal fluoroquinolone resistance mutations in the human gut microbiota changes after travelling from Sweden, a country with low fluoroquinolone consumption and resistance, to India, a country with relatively high consumption and resistance. The significant increase in the S83L substitution in GyrA – one of the target proteins for quinolones – shows that the *Escherichia* communities residing in the gut of the Swedes were altered during their stay in India in a direction towards a higher resistance potential. Furthermore, travel was associated with a significant reduction in the number of observed genotypes. Hence, this study illustrates that travel not only contributes to the spread of mobile antibiotic resistance genes but could also promote the spread of bacteria carrying chromosomal resistance mutations.

It is plausible that the increase in resistance mutations is the result of local strains, obtained from e.g. Indian water and food [26], colonizing the gut of the Swedish study participants. Opportunities for changes in the gut microbiome while travelling are indeed ample. All but one of the Swedish participants (individual 193) suffered from traveller’s diarrhoea – a very common fate for Swedes visiting India [27] – and such a disturbance of the flora could provide niches that are easily filled by indigenous bacterial strains. Alternatively, the bacteria causing the diarrhoea could be carrying the resistance mutations. Indeed, the only Swede in this study that eluded diarrhoea showed a moderate decrease in the abundance of the S83L substitution. The correlation between antibiotic resistance and diarrhoea has also been suggested in a study that showed that diarrhoea during travel was associated with a higher risk of acquired ESBL-producing Enterbacteriacea into to the faecal flora [5]. Alas, with the approach taken, we cannot exclude that various changes in other factors, such as environment and food habits [28, 29], could have provided selective advantages for pre-existing strains in the gut of the Swedes. The Swedish participants in this study did, however, not undergo treatment with any antibiotics during their visit in India. Thus, there was no apparent selection pressure that would favour pre-existing strains carrying antibiotic resistance mutations. Consequently, the acquisition of new strains seems like a more plausible explanation. A future research aim could be to elucidate the geographical origin of resistant *Escherichia* strains by employing culture methods and high-resolution genetic strain characterization by, for example, sequencing of multiple genomic regions. If the participants had taken antibiotics during their travel, it is possible that the observed effect would have been stronger.

The most abundant substitutions in the study were S83L and D87N in GyrA and S80I in ParC, all of which have been previously linked to fluoroquinolone resistance. In fluoroquinolone-resistant clinical isolates, S83 is the most common amino acid in GyrA to be substituted, S83L is the most common substitution, and D87N is the second most common substitution [10]. As single mutations, neither substitution provides *E. coli* with clinically relevant ciprofloxacin resistance (increase in ciprofloxacin MIC from 0.01 mg/L to 0.38 mg/L and 0.25 mg/L, respectively), nor are they associated with any known significant fitness cost in competition experiments with a wild-type strain [30]. In contrast, a double substitution of S83L and D87N provide the same protection against quinolones as the single S83L substitution but also introduce a fitness burden (measured relative fitness of 0.97) [30]. In ParC, S80 is the most commonly substituted amino acid, and S80I is the most commonly detected substitution. Having only the single S80I substitution in ParC is not associated with either an increased ciprofloxacin MIC or a fitness cost. However, the small fitness cost associated with S83L/D87N in GyrA is eliminated if ParC acquires the S80I substitution (measured relative fitness of 1.01) [30]. This provides a plausible explanation for the correlation between the abundance of the two genotypes (Fig. 3). Alas, with the metagenomic amplicon sequencing approach used on the two genes in this study, it is impossible to determine which of the GyrA alterations occurred in the same bacterium as the ParC alterations. However, the detected correlation, together with the previously reported reduced fitness cost, suggests that these alterations, at least to a large extent, co-occur in the same bacteria. In addition to eliminating the fitness cost for S83L/D87N double mutants, the S80I substitution also increases the ciprofloxacin MIC to 32 ml/L, which is well above the EUCAST breakpoint for clinical resistance in Enterobacteriaceae (1 mg/L) [31].

It is thought that the majority of urinary tract infections caused by *E. coli* have their origin from the patient’s own gut flora [32]. Therefore, one could hypothesise that there could be a correlation between the average abundance of resistance mutations in the gut microbiota and the frequency of fluoroquinolone-resistant *E. coli* infections encountered in the clinic. The combination of S83L in GyrA and S80I in ParC has been reported to result in clinical resistance (ciprofloxacin MIC 1 mg/L) but it is not associated with a fitness cost [30]. Assuming that all observed S80I substitutions in ParC occur in bacteria carrying the S83L substitution in GyrA, the theoretical maximum proportion of the examined *Escherichia* gut communities being clinically resistant to fluoroquinolone through known chromosomal target gene mutations would on average be 16 % in the Swedish samples taken prior to travel and 21 % in the Indian samples. This does not reflect the frequency of fluoroquinolone resistant infections, which in Sweden was 7.9 % of invasive *E. coli* infections [14], and in India could have been as high as 73 % of uropathogenic *E. coli* [16]. It should be stressed that the present study did not cover representative populations of either countries, as it was mainly designed to examine the effect of travel. However, the data indicate that the abundance of resistance mutations in the entire *Escherichia* community residing in the gut differs from the pathogenic portion.

The finding of a decreased number of genotypes after travel was intriguing. We have not encountered any studies showing that travel effects the intestinal bacterial diversity or composition. Metagenomic sequencing of the Swedish samples included in this study in addition to more samples of Swedish travellers, has shown that the overall taxonomic composition remains relatively stable [33]. However, when only the small proportion of the community composing of the *Escherichia/Shigella* genera was considered, travel resulted in an on average four-fold increase in the genus abundance (average abundance of *Escherichia/Shigella* was 0.018 and 0.084 % before and after travel, respectively). Taken together, the increased abundance of *Escherichia*/*Shigella* and decreased number of observed genotypes of the QRDRs of *gyrA *and *parC*, show that the faecal flora of the Swedish travellers comprised of a larger but less variable *Escherichia*/*Shigella* population after return from India. This population also has a higher abundance of quinolone resistance mutations.

## Conclusions

Our results indicate that international travel can be associated with increased abundance of faecal bacteria carrying chromosomal resistance mutations. Thereby, traveling may contribute to the global dissemination of *Escherichia* carrying chromosomal mutations linked to quinolone resistance. Furthermore, the high abundance of genotypes encoding GyrA and ParC with amino acid substitutions previously linked to quinolone resistance, suggest that these particular alterations are not associated with a fitness cost in the studied environment. Amplicon sequencing of metagenomic DNA proved to be a comprehensive method to study resistance mutations in an community of bacteria without the requirement of culturing.

## Availability of supporting data

The raw sequence data have been made available in the Sequence Read Archive (SRA) connected to BioProject accession number PRJNA241337.

## Additional file

Additional file 1: Table S1. Metadata and numbered of analysed reads for each sample. **Table S2.** Additional metadata of Swedish study participants. **Table S3.** Average abundance of amino acid substitutions detected in >1 % of all reads. (DOC 91 kb)

## Abbreviations

DDD: Defined daily doses
ESBL: Extended-spectrum β-lactamase
QRDR: Quinolone-resistance determining region
