# Supplementary material for: Quinolone resistance mutations in the faecal microbiota of Swedish travellers to India
Source: BMC Microbiol. 2015 Oct 24;15:235. doi: 10.1186/s12866-015-0574-6 (PMC4619388; doi:10.1186/s12866-015-0574-6)
Supplement: Additional file 1: Table S1. — Metadata and numbered of analysed reads for each sample. Table S2. Additional metadata of Swedish study participants. Table S3. Average abundance of amino acid substitutions detected in >1 % of all reads. (DOC 91 kb) [file 12866_2015_574_MOESM1_ESM.doc]

# Supplemental materials

| **TABLE S1** Metadata and number of analysed reads for each sample | **Abundance of most common substitutions*** | **ParC** | **80I** | 14% – 3% | 34% – 2% | 6% – 17% | 6% – 0% | 21% – 74% | 7% – 1% | 38% – 5% | 36% – 1% | 28% – 1% | 1% – 3% | 3% – 2% | 2% – 3% | 6% | 2% | 2% | 6% | 0% | 8% | 22% | 93% | 68% | 5% | 6% | 34% | *All substitutions detected in >5% of all analysed reads are included. Swedes: sample collected before travel to India – Sample collected after returning to Sweden |
| --- | --- | --- | --- | --- | --- | --- | --- | --- | --- | --- | --- | --- | --- | --- | --- | --- | --- | --- | --- | --- | --- | --- | --- | --- | --- | --- | --- | --- |
|  |  | **GyrA** | **83L + 87N** | 23% – 47% | 28% – 2% | 3% – 17% | 6% – 0% | 23% – 80% | 24% – 2% | 26% – 2% | 15% – 0% | 10% – 1% | 0% – 4% | 1% – 7% | 17% – 3% | 2% | 1% | 1% | 6% | 99% | 54% | 1% | 92% | 82% | 1% | 6% | 30% |  |
|  |  |  | **87N** | 23% – 47% | 28% – 2% | 3% – 17% | 7% – 0% | 23% – 80% | 24% – 3% | 26% – 12% | 15% – 0% | 11% – 1% | 0% – 4% | 1% – 7% | 18% – 3% | 2% | 1% | 1% | 6% | 100% | 54% | 1% | 93% | 82% | 1% | 6% | 30% |  |
|  |  |  | **83L** | 60% – 89% | 65% – 48% | 5% – 98% | 18% – 46% | 45% – 86% | 50% – 22% | 62% – 83% | 43% – 84% | 40% – 95% | 0% – 96% | 63% – 55% | 75% – 61% | 35% | 54% | 74% | 18% | 100% | 89% | 40% | 98% | 91% | 10% | 29% | 79% |  |
|  | **Number of analysed reads** | **ParC** | **After** | 22 986 | 19 610 | 17 302 | 16 441 | 20 295 | 14 971 | 15 755 | 13 853 | 17 030 | 12 452 | 12 188 | 11 669 | 9 015 | 9 009 | 12 145 | 13 082 | 10 658 | 9 882 | 12 553 | 11 894 | 14 357 | 8 368 | 8 061 | 10 076 |  |
|  |  |  | **Before** | 21 875 | 15 099 | 14 138 | 16 529 | 14 014 | 18 352 | 14 364 | 12 265 | 13 857 | 11 031 | 11 365 | 11 763 |  |  |  |  |  |  |  |  |  |  |  |  |  |
|  |  | **GyrA** | **After** | 5 263 | 6 058 | 6 711 | 8 043 | 6 628 | 6 260 | 5 348 | 9 096 | 4 637 | 5 227 | 3 228 | 5 719 | 4 003 | 4 227 | 4 103 | 2 677 | 6 040 | 3 157 | 6 904 | 6 011 | 4 578 | 1 830 | 1 300 | 4 214 |  |
|  |  |  | **Before** | 4 185 | 3 721 | 7 115 | 6 783 | 4 967 | 5 858 | 4 520 | 728 | 3 461 | 5 283 | 4 842 | 5 318 |  |  |  |  |  |  |  |  |  |  |  |  |  |
|  | **Sex** | | | M | F | M | F | F | F | F | F | M | F | F | F | M | M | F | F | M | M | F | F | M | M | M | M |  |
|  | **Age** | | | 24 | 25 | 23 | 20 | 29 | 30 | 23 | 23 | 24 | 27 | 28 | 28 | 18 | 26 | 29 | 21 | 30 | 12 | 13 | 32 | 15 | 34 | 18 | 40 |  |
|  | **Individual** | | | 103 | 120 | 121 | 125 | 139 | 146 | 154 | 170 | 176 | 177 | 193 | 221 | V 1 | V 23 | VI 11 | VI 15 | VI 7 | VII 10 | VII 14 | VII 5 | VIII 12 | VIII 14 | VIII 19 | VIII 22 |  |
|  |  | | | Sweden | | | | | | | | | | | | India | | | | | | | | | | | |  |

| **TABLE S2** Additional metadata of Swedish study participants | | | | | |
| --- | --- | --- | --- | --- | --- |
| Individual | Days sampling – departure | Days return – sampling | Days in India | Main destination | Patient contact |
| 103 | 2 | 5 | 36 | Bangalore | Yes |
| 120 | 23 | 44 | 29 | Bangalore | Yes |
| 121 | 4 | 25 | 98 | Unknown | No |
| 125 | 4 | 37 | 98 | Bangalore | No |
| 139 | 1 | 2 | 38 | New Delhi, Goa | Yes |
| 146 | 18 | 33 | 28 | Unknown | Yes |
| 154 | 77 | 15 | 106 | Bangalore | Yes |
| 170 | 0 | 19 | 105 | Bangalore | No |
| 176 | 16 | 20 | 32 | Unknown | Yes |
| 177 | 1 | 25 | 43 | Unknown | No |
| 193 | 7 | 32 | 58 | Bangalore | Yes |
| 221 | 9 | 4 | 92 | Bangalore | Yes |

| **TABLE S3** Average abundance of amino acid substitutions detected in >1% of all reads | | | | | |
| --- | --- | --- | --- | --- | --- |
| **Gene** | **Substitution** | **Swedes** | | **Indians** | **Quinolone resistance**^19^ |
|  |  | **Before travel** | **After travel** |  |  |
| GyrA | 83A | 0.14% | 0.67% | 4.4% | Yes |
|  | 83L | 41% | 72% | 67% | Yes |
|  | 83T | 2.5% | 0.30% | 0.32% | No |
|  | 87G | 1.1% | 0.26% | 4.4% | Yes |
|  | 87N | 14% | 15% | 38% | Yes |
|  | 112V | 2.8% | 0.38% | 0.44% | No |
| ParC | 80I | 17% | 11% | 24% | Yes |
|  | 80R | 0.31% | 0.076% | 8.6% | Yes |
|  | 84K | 0.69% | 0.13% | 11% | Yes |
|  | 84V | 2.2% | 0.16% | 1.3% | Yes |
